# School-aged children based seasonal malaria chemoprevention using artesunate-amodiaquine in Mali

**DOI:** 10.1016/j.parepi.2018.02.001

**Published:** 2018-02-07

**Authors:** Mahamadou A. Thera, Abdoulaye K. Kone, Bourama Tangara, Elizabeth Diarra, Sirama Niare, Abdramane Dembele, Mahamadou S. Sissoko, Ogobara K. Doumbo

**Affiliations:** a Malaria Research and Training Centre-International Center for Excellence in Research (MRTC-ICER), Department of Epidemiology of Parasitic Diseases, Faculty of Medicine and Dentistry, USTTB, Point G, BP 1805 Bamako, Mali; b Service of Psychiatry, University and Hospital Center of Point G, Bamako, Mali

**Keywords:** School-aged children, Artesunate–amodiaquine (ASAQ), Seasonal malaria chemoprevention (SMC), School-based interventions, Malaria elimination, Peri-urban, Mali

## Abstract

**Introduction:**

Malaria is still a public health problem in Africa. Seasonal Malaria Chemoprevention (SMC) is an efficient control strategy recommended by WHO that targets children under five year old living in areas of seasonal malaria transmission. SMC uses the combination amodiaquine (AQ) – sulfadoxine-pyrimethamine (SP). However SP selects rapidly drug resistant parasites. And malaria burden may increase in older children where SMC is implemented. We initiated a pilot study to assess an alternative approach to SMC in older children in Mali.

**Methods:**

A randomized open-label clinical trial was conducted to test the efficacy and safety of SMC using artesunate – amodiaquine in school aged children in Mali. Two hundred pupils aged 6–15 years old were enrolled and randomized into two arms of 100 each, to receive either artesunate–amodiaquine (ASAQ) monthly or no intervention. Both arms were followed and clinical malaria were diagnosed and treated with arthemeter-lumefanthrine as recommended by Mali National Malaria Control Program. ASAQ was administered 3 days under study team direct observation and during 4 consecutive months starting in October 2013. Follow up was continued until April 2014.

**Results:**

Overall, 20 cases of uncomplicated clinical malaria were encountered in the Control arm and three cases in the ASAQ arm, showing a protective efficacy of 85% 95% CI [80.1–89.9] against clinical malaria. Protective efficacy against malaria infection was 69.6% 95% CI [58.6–21.4]. No effect on anemia was observed. ASAQ was well tolerated. Most common solicited adverse events were abdominal pain and headaches of mild intensity in respectively 64% and 44% of children that swallowed ASAQ.

**Conclusion:**

ASAQ is effective and well tolerated as SMC targeting older children in a *peri* urban setting in Mali. Its administration at schools is a feasible and accepted strategy to deliver the intervention.

## 1 Introduction

Despite tremendous reduction in the burden of malaria in the last decade thanks to control efforts, malaria remains a public health problem in most malaria endemic countries and accounted for 445,000 deaths in 2016, 91% of which occurred in sub Saharan Africa (WHO, 2017). Seasonal malaria chemoprevention (SMC) is an effective control strategy specifically recommended by WHO in April 2012, for countries where >60% of the burden of malaria occurred in the three months of the rainy season, that coincides with the malaria transmission season (WHO, 2012). SMC targets children aged under five years and consists of administering a single curative dose of Sulfadoxine-Pyrimethamine (SP) associated with a three-day course of amodiaquine (AQ). The combination treatment is given once a month during 3 months with the aim to prevent malaria during the transmission season (WHO, 2012). Evidence in several African countries has shown that SMC using SP-AQ is highly efficacious, eradicating almost severe malaria and leading to strong reduction in *P*. *falciparum* prevalence, in the incidence of clinical uncomplicated malaria and malaria anemia (Dicko et al., 2011a; Konaté et al., 2011; Odhiambo et al., 2010; Bojang et al., 2011). However large-scale implementation of SMC has been associated with selection of Pfdhfr-dhps quintuple mutant genotype (Maiga et al., 2016). Furthermore, administration of SP during pregnancy has been associated with selection of parasites carrying genetic quintuple mutations (Pfdhfr 51I, 59R, and 108 N; Pfdhps 437G and 581G) (Conrad et al., 2017). The quintuple mutation is known to be associated with falciparum parasite resistance to SP. Therefore, in places where SMC is implemented using SP, a critical recommendation is to monitor closely the level of circulating parasites sensitivity to SMC drugs. In addition, more studies are encouraged to find alternative treatment regimens to SP-AQ. One alternative regimen constitutes the use of a highly efficacious artemisinine (AS) derivative in association with AQ. Artesunate is a soluble derivative quickly adsorbed orally, the highest concentration in blood is achieved 1 h following an oral intake and the half-life is between 20 and 72 min (Orrell et al., 2008; Saunders et al., 2012; Matar et al., 2014). AS pharmacokinetics parameters are similar when AS is given alone and when AS is combined with AQ (Orrell et al., 2008). Artesunate (AS) is highly efficacious in West Africa. Efficacy studies done *in vivo* have not detected any slowness in parasite clearance time (Maiga et al., 2012). AS has been assessed in combination therapies with AQ (ASAQ) in community-based studies in Ghana under a SMC regimen in children aged 6–60 months (Ahorlu et al., 2011). ASAQ reduced parasite carriage rate by 90.0%, and the prevalence of anemia by 43.1%. In addition the prevalence of fever was reduced by 85.0% (Ahorlu et al., 2011; Kweku et al., 2008). A similar efficacy pattern was reported in a trial done in Kenya where ASAQ was compared to SP-AS in children aged <5 years (Odhiambo et al., 2010). ASAQ stands therefore as an interesting alternative SMC regimen in case resistance to SP would arise in West Africa.

Areas of seasonal malaria transmission in West Africa constitute the places where SMC is the most cost-effective approach. These are also the places where decrease in malaria burden is being reported (WHO, 2017) associated with a shift in the disease burden to include older children, in particular school-aged children (Nankabirwa et al., 2014a). There is no control intervention that specifically target school-aged children. Moreover, where SMC is scaled-up, the overall reduction in malaria burden is associated with an increase in malaria morbidity in older children (Nankabirwa et al., 2014a). Therefore, specific interventions targeting school-aged children are urgently needed.

In this pilot study we have evaluated the impact of SMC using four doses of ASAQ at one-month intervals in school-aged children at the periphery of Bamako, the capital city of Mali. We aimed to assess the impact of the intervention on malaria morbidity. We also aimed to assess the delivery of SMC strategy using schools as a modality to have access to a large well characterized target population.

## 2 Materials and methods

This was an open-label, randomized controlled clinical trial designed to assess the efficacy and safety of SMC using ASAQ in school-aged children.

### 2.1 Study setting

The study was conducted in Sirakoro-Meguetana, a setting of 15,000 inhabitants located at the southeastern suburbs of Bamako, the capital city of Mali. Malaria transmission is low, seasonal from July to December and annual rainfall ranges between 700 and 1300 mm per year. Malaria prevalence is not known in Sirakoro-Meguetana. However, the setting is similar to Sotuba, another *peri* urban area located in the northern suburbs of Bamako. In Sotuba malaria transmission is variable from one year to another, depending on the level of rainfall. With abundant rainfalls, during the rainy season, malaria prevalence in children aged <15 years, can reach 15.1% (*n* = 1142) (Sissoko et al., 2015). While in years with lower annual rainfall malaria prevalence is limited to 8% (*n* = 171) (Dicko et al., 2007). The village hosts a large scholar complex composed of five schools offering primary and secondary education with 4000 pupils recorded in 2013. Close to the school a community based health care center provides curative and preventive care to Sirakoro-Meguetana community members under the leadership of a medical doctor.

### 2.2 Participants

Participants were children aged 6 to 15 years attending the fundamental school in Sirakoro-Meguetana. After a screening for eligibility, they were included if they agreed to comply with study follow up procedures, had no clinical signs and symptoms of danger, had no known allergic reaction to amodiaquine and artesunate, and if their parents or legal guardians gave a written informed consent. Exclusion criteria were the presence of a low hemoglobin level (<10 g/dL), a low blood sugar level (<70 mg/dL) or a positive malaria smear at screening, the presence of any concurrent acute illness, the presence of chronic illnesses such as diabetes or hypertension, the intake of any antimalarial drugs within 15 days preceding the screening.

### 2.3 Ethical clearance

The Institutional Ethics Committee from the faculty of Medicine, Pharmacy and Odonto-Stomatology approved the study protocol and informed consent forms, approval letter 2013/76/CE/FMPOS dated on 31 July 2013. Community permission was sought as described (Diallo et al., 2005) from local administrative, health, traditional authorities as well as from the direction of the school of Sirakoro-Meguetana. The study was conducted in conformity with the International Conference on Harmonization Good Clinical Practices, the Declaration of Helsinki and applicable regulatory requirements of Mali. The clinical trial was registered in a public registry, the Pan African Clinical Trial Registry (PACTR), under the number PACTR201409000782309.

### 2.4 Interventions and participants follow up

The study drugs are marketed in Mali and freely available. Artemisinin-based combination therapy (ACT), composed of artesunate (AS) and amodiaquine (AQ), so-called ASAQ is one the of first line drug recommended by World Health Organization and the Mali National Malaria Control Program (MNMCP) to treat uncomplicated malaria. Artemether-Lumefantrine, abbreviated as ARLU is the other ACT recommended by MNMCP. These drugs are widely available in Mali and distributed free of charge in the public health care system to children aged under 5 years. ASAQ used in this study was manufactured under GMP conditions by Guilin Pharmaceutical Co., Ltd., and donated solely to the purpose of the study.

Children in the study arm under SMC received tablets of ASAQ that they swallowed under direct supervision by study team on study day 0, 1 and 2. After drugs intake, children remained 1 h under study team surveillance. Adverse reactions after drugs intake as well as concomitant medications were recorded. If a child vomited <30 min after his oral intake, a full dose was given. When vomiting occurred after 30 mn and up to 1 h after drug administration, half dose was given. The first dose of ASAQ was given to all eligible children in October 1st, 2013. Subsequent doses were given one month apart; the second dose in November 2013, the third dose in December 2013 and the fourth dose in January 2014.

In the control arm, no product was administered. Children were followed as recommended by MNMCP and whenever they had signs or symptoms they were fully examined for diagnosis and treatment. In both arms, clinical episodes of malaria were diagnosed using a Rapid Diagnostic Test (RDT), the SD Bioline Malaria Ag Pf/Pan (Djallé et al., 2014). Malaria smears were also confectioned and read at the end of the follow up period. Children with RDT-confirmed clinical malaria were treated using the ARLU regimen recommended by the MNMCP. Children in both arms were all followed during six months until April 30th, 2014. Follow up was active and passive. Under the passive follow up, the study team was present in permanence in the school for treatment of any incident cases of clinical malaria or others ailments at any time during the study period. All illnesses that occurred were diagnosed and treated by the study team or referred to appropriate health care centers as per national procedures. Under the active follow up, a scheduled visit of all enrolled children was done every 4 weeks. During the scheduled visits, a complete clinical examination of the children was done, blood was collected for determination of hemoglobin level and confection of a malaria smear. Hemoglobin was determined using Hemocue® HB201 DM system. The malaria smears were Giemsa-stained as described (Makler et al., 1998; Coulibaly et al., 2014), and also read at the end of the follow up.

### 2.5 Outcomes

The primary outcome was the efficacy of the intervention. This was determined by comparing the incidence of clinical malaria between the ASAQ arm and the control arm. It was also determined by comparing the incidence of malaria infection and anemia between the two study arms. The secondary outcome was the safety of ASAQ determined as incidence of adverse events after oral intake of ASAQ tablets.

#### 2.5.1 Efficacy assessment

Clinical malaria was the primary endpoint and was defined as the presence of clinical symptoms consistent with malaria such as fever, headaches, abdominal pain, diarrhea, vomiting, or myalgia, associated with the presence of asexual forms of *P*. *falciparum* on malaria smear, in the absence of signs of severity or complications. School teachers were trained to detect children presenting signs or symptoms and to refer them the study team. In addition, children were also informed regularly to self-refer to the study team whenever they would feel ill. Malaria infection was defined as the presence of asexual forms of *P*. *falciparum* on malaria smear without associated clinical signs or symptoms. These cases were identified after the end of the follow up period when smears collected at schedule visits were read. Hemoglobin rate was determined with Hemocue® on capillary blood. Moderate anemia was defined as a hemoglobin rate equal or below 10 g/dL and severe anemia was defined as hemoglobin rate equal or below 05 g/dL. A questionnaire asking for use of bed nets impregnated with insecticides and status of others socio-economic variables was used to interview enrolled children at baseline.

#### 2.5.2 Safety assessment

After each administration of ASAQ, participants were monitored for 1 h, during which, occurrence of solicited adverse events was determined on study days 0, 1 and 3. Solicited adverse events were vomiting, nausea, abdominal pain, fever (oral temperature ≥ 37,5 °C), headache, anorexia, diarrhea, myalgia, dizziness, and pruritis. All others adverse events were considered as unsolicited adverse events. Adverse events were evaluated for relationship with ASAQ oral intake and graded by intensity into four classes. Adverse events were of: class 1, if they were mild with signs and symptoms easily tolerated; class 2, if they were moderated with intensity high enough to disturb daily activities; class 3 if they were severe, with invalidating intensity impeding daily activities; class 4 if they were life threatening.

### 2.6 Sample size

Sample size was determined based on the primary endpoint, the incidence of clinical malaria. The primary analysis population was the intent-to-treat (ITT) population. Power calculations were based on the inverse sine transform approximation (Meinert, 2012). With a sample size of 100 participants in each arm, the study has a power of 80% to detect a difference of 50% in the incidence of clinical malaria between the two arms, assuming an incidence of 0.30 clinical episodes per child and transmission season in the control arm, a type I risk of error of 5% and a 5% lost to follow up rate.

### 2.7 Randomization

Participants were randomized in a 1:1 ratio to receive either ASAQ or no intervention. Randomization to study arms was done using a computer-generated randomization list. The randomization list contained sequential codes that linked a study number to an arm assignment. Study numbers were assigned to children in the order in which they were enrolled in the trial. There were no masking procedures of study products assignments.

### 2.8 Data analysis

The trial was conducted in compliance with the Bandiagara Malaria Project (BMP) Clinical Trial Quality Assurance Procedures and the MRTC Clinical Laboratory Quality Assurance Plan. The sponsor ensured three monitoring visits of the study. After the pre-study visit, one interim monitoring visit and a close-out visit were done. Data were collected on standard case report forms that were monitored and sent to MRTC Data Management Unit for data entry into a GCP compliant database designed with Microsoft ACCESS 2007. Data was double entered by two independent data entry clerks and then reconciled by a senior data manager. After resolution of discrepancies, the database was frozen and used by the study statistician for analysis.

### 2.9 Statistical methods

Categorical data are summarized using counts and percentages. For efficacy analysis, incidence of clinical malaria and proportion of malaria infection episodes between study arms were compared using Chi square test and relative risk ratios. Parasite density and anemia were analyzed as binary variables using Chi square test and also as continuous variables using ANOVA after appropriate logarithmic transformation. Survival analysis was done to assess difference in time to first or only clinical malaria episodes between the two arms. The time to first clinical episode of malaria or malaria infection was analyzed using a Kaplan–Meier curve and the Log rank test. All tests were 2-sided, and no correction of *p*-values was made for additional analyses.

## 3 Results

### 3.1 Participant flow and baseline data

**Fig. 1:**
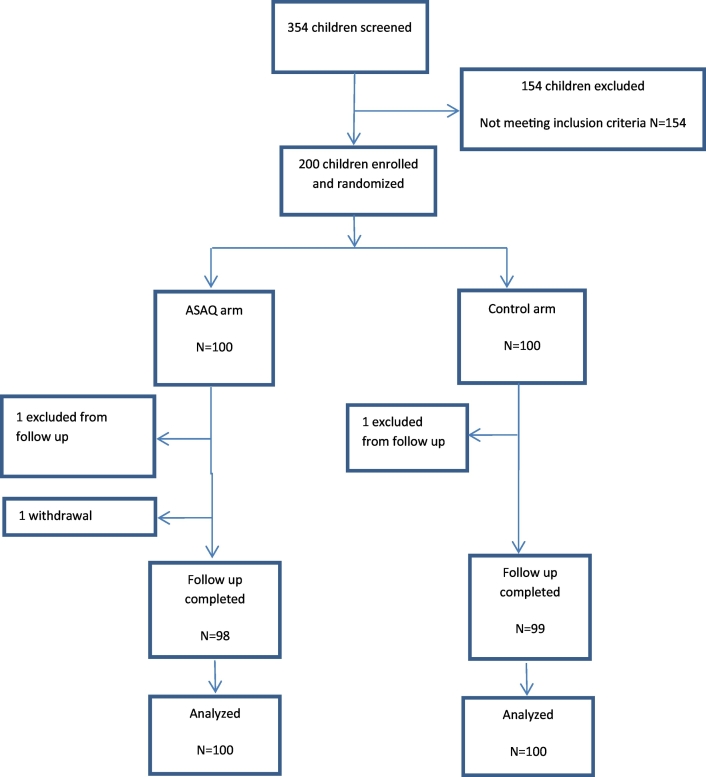
Trial profile.

Three hundred fifty-four children were screened and 200 were randomized, 100 in each study arm. Main reasons for exclusions were concurrent illnesses (Fig. 1). The mean age was 9.8 years and the sex ratio male/female was 0.83. Participants were comparable at inclusion with regard to baseline characteristics such as mean age, parasite density geometric means, hemoglobin level, use of impregnated bed nets and parent literacy or wealth, appreciated by the presence of house electricity (Table 1). In the ASAQ arm, two participants were excluded after randomization; one because of an adverse event due to ASAQ and reported by his father and the second for non-compliance with study visits schedule. In the control arm, one participant was excluded for non-compliance with the study visits schedule.

**Table 1:** Baseline characteristics.

Characteristics	ASAQ *N* = 100	Control *N* = 100	*p* value
Mean age in year ± SD	9.73 ± 0.22	9.79 ± 0.24	ns
Female (%)	56.0	53.0	ns
Declared use of impregnated bed nets (%)	42.0	45.0	ns
House electrified (%)	65.0	64.0	ns
Mean Hemoglobin (g/dL) [95% CI]	12.0 [11.8–12.2] (*n* = 99)	11.9 [11.8–12.2] (*n* = 98)	ns
Parasite density geometric mean [95% CI]	1400.5 [60.4–32,481.5] (*n* = 5)	1372.6 [384.3–4903.2] (n = 10)	ns

CI = confidence Interval, ns = non-significant, SD = standard deviation.

### 3.2 Efficacy

#### 3.2.1 Efficacy against Plasmodium falciparum

**Fig. 2:**
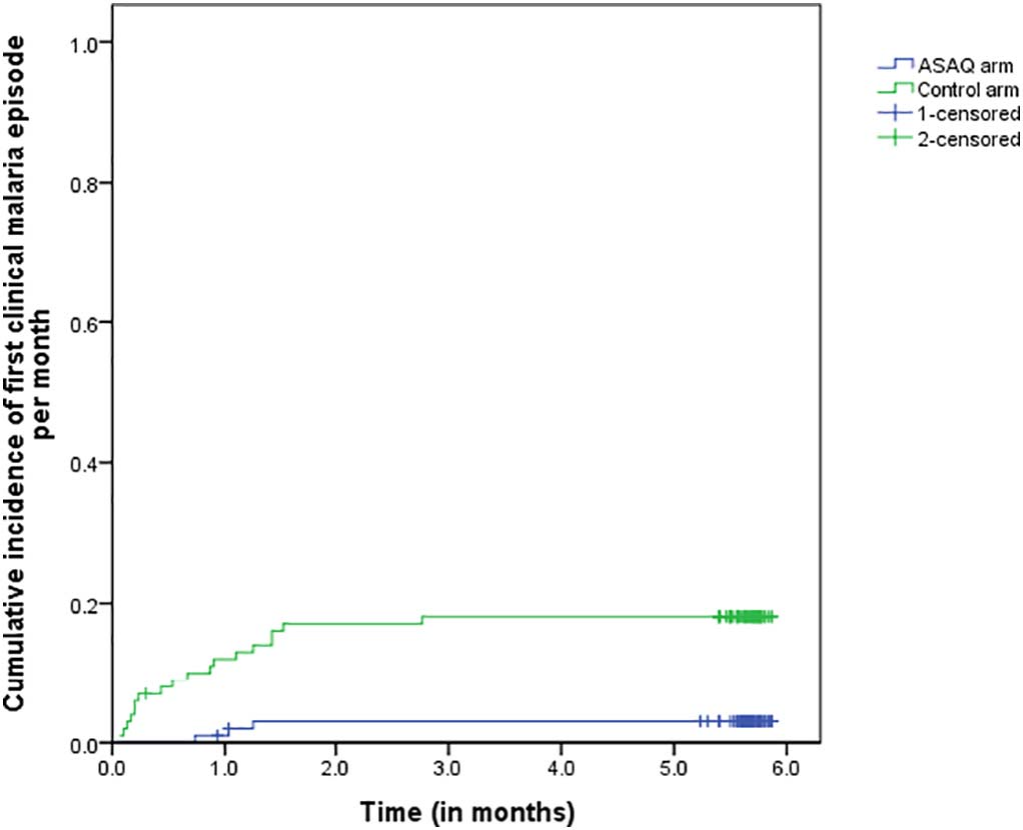
Protective efficacy of ASAQ against clinical malaria. *Log Rank p* = *0*.*001*. 0.0 = October 2013, 1.0 = November 2013, 2.0 = December 2013, 3.0 = January 2014, 4.0 = February 2014, 5.0 = March 2014, 6.0 = April 2014.

In the ASAQ arm, three participants had each, one clinical episode of malaria as defined for the primary endpoint among the 100 children followed for 6 months and a total of 557.9 person-months. In the control arm, 18 children had 20 clinical episodes of malaria recorded among the 100 children followed during 6 months with a total person month of 565. Cumulative incidence of clinical malaria was 5.377.10^−3^ per person month in the ASAQ arm and 35.98.10^−3^ per person month in the control arm. Relative Risk of having a clinical malaria in the control arm when compared to the ASAQ arm was 6.58 CI 95% [1.95–22.15]. Protective efficacy of the SMC ASAQ compared to control was 85% CI 95% [80.1–89.9] against clinical malaria (Table 2). The Kaplan–Meier curve showed that no clinical malaria episode occurred in the ASAQ arm after the second month of drug administration corresponding to November 2013. While a clinical malaria episode was encountered in January 2014 in the control arm (Fig. 2).

**Table 2:** Impact of ASAQ on clinical malaria and malaria infection.

Characteristics	ASAQ *N* = 100	Control N = 100
Episodes of clinical malaria	3	20
Person time at risk (in months)	555.9	565
Cumulative incidence clinical malaria/month	0.0053	0.0353
RR of clinical malaria [95% CI]	6.58 [1.96–22.15]	
Protective efficacy against clinical malaria (%, [95% CI])	85.0% [80.1–89.9]	
Episodes of malaria infection	7	23
Parasite density geometric mean^a^	601.02	892.83
RR of malaria infection [95% CI]	3.24 [1.39–7.56]	
Protective efficacy against malaria infection (%, [95% CI])	69.6 [58.6–71.4]	

a *ANOVA to compare parasite density geometric means between the two arms*, *p* = *0*.*35*.

A Relative Risk of 3.24 CI 95% [1.39–7.56] of being infected in the control arm as compared to the ASAQ arm is reported. Protective efficacy against malaria infection was 69.6% 95% CI [58.6–71.4]. Highest parasite density was 33,300 trophozoïtes per microliter, observed in the ASAQ arm. There was no statistically significant difference in the parasite density geometric means between the two arms (Table 2).

#### 3.2.2 Efficacy against anemia

In the ASAQ arm, 11 children developed anemia with hemoglobin below 10 g/dL during the follow up. In the control arm, 19 cases of anemia were recorded. The difference between the two arms was not statistically significant. No episodes of severe anemia were recorded.

### 3.3 Safety

Most common solicited adverse events were abdominal pain reported by 64% of children that received ASAQ, headaches reported by 44% of children, dizziness reported by 22% of children, nausea reported by 7% of children and vomiting reported by 6% of children. Mean duration of adverse events were 2.8 days for abdominal pain, 3.3 days for headaches, 3.8 days for dizziness, 1.7 days for nausea, 1.4 days for vomiting. Anorexia was reported by 3% of children and lasted 7.5 days the longest duration an adverse event was reported (Table 3). These signs and symptoms were reported shortly after the first administration and were all of mild intensity. These solicited events occurred also at the second, third and fourth administration of ASAQ with declining frequencies. These events were also solicited in the Control arm, where mainly abdominal pain and headaches were reported. Most common unsolicited adverse events were Acute Respiratory Infections (ARI) and dental pain observed in similar proportion in both study arms (Table 3). The unsolicited events were not related to ASAQ administration.

**Table 3:** Solicited and unsolicited adverse events in the ASAQ and Control study arms.

Adverse events	ASAQ (N = 100)	Control (N = 100)	Mean duration (in days)
Solicited adverse events			
Abdominal pain	63	24	2.8
Headaches	46	33	3.3
Dizziness	23	0	3.8
Nausea	8	0	1.7
Vomiting	7	0	1.4
Pruritis	2	0	4.0
Anorexia	3	0	7.5
Fever	2	4	3.4
Diarrhea	1	1	1.5
Myalgia	1	0	4.0

Unsolicited adverse events			
ARI (acute respiratory infection)	43	45	7.4
Dental pain	5	6	5.4
Ear-nose-throat	1	6	6.3
Injury	2	6	5.0
Others	12	12	5.5

## 4 Discussion

This pilot study showed that ASAQ was highly effective as a SMC strategy against both clinical malaria and malaria infection determined by thick smear when administered at a *peri* urban school children aged 6 to 15 years old in Mali.

We enlisted school teachers and obtained their help in maintaining contact with pupils. Such close partnership established with the school teachers, allow us to be confident that all cases of illnesses that occurred during the study were captured. School teachers have been successfully involved in community-based approach to deliver malaria control intervention (Lalji et al., 2016).

The study setting is located around Bamako, an area of low malaria transmission with seasonal peaks during rainy season. The study sample size was powered to detect a diminution by half of malaria incidence assuming a 30% incidence in the control arm. We observed a lowered than anticipated incidence of malaria. Only 23 clinical episodes of malaria were recorded. However most of the disease occurred in the control group, showing an 85% of protective efficacy. This is even more remarkable, given that the intervention started in October, when rainfall is ending, while uncomplicated malaria is not increasing up. It's noteworthy to highlight that even in such conditions SMC is still highly effective.

This result should however be examined cautiously in the light of the study limitations that must be considered. First element is the study design which was open label. Data collection bias could have derived from investigators assessing study endpoints, as well as teachers that helped in interactions with the pupils in their respective classrooms, or children themselves who might have behave differently in relation with their study assignments. The randomization has allowed us to constitute two groups similar at baseline. The study has been conducted respecting strictly ICH/GCPs guidelines. The study oversight done by senior investigators encouraged the study team to minimize bias related to participants follow up. A second limitation is the diagnosis of clinical malaria using only RDT. At the end of the follow up all malaria smears were read, and the results of smears are reported. Submicroscopic malaria importance has been recognized (Manjurano et al., 2011). This limit is less critical because missing submicroscopic parasites would not have a negative influence on the ASAQ efficacy.

Classically SMC is administered at the beginning of the transmission season. We have administered our first round in October in an area where transmission season starts in July. Studies in sites with similar highly seasonal patterns of malaria transmission, like in Bandiagara (Coulibaly et al., 2013; Coulibaly et al., 2017) have shown that malaria incidence peaks one month after the peak of the rainfall. In our study setting, schools were in holidays in the period of peak transmission covering the months of August and September, period during which most pupils are not reachable. Despite our late start, we still observed a very high protective efficacy against clinical malaria as well as infection due to *P*. *falciparum*. Such efficacy is observed on the top of others malaria control interventions routinely implemented. But we should note that even if our study participants were encouraged to use impregnated bed nets, when interviewed, only 45% have declared that they did use the bed nets.

Previous studies of SMC have targeted children much younger than the pupils we have enrolled. The increased burden of malaria reported in this population (Nankabirwa et al., 2014a) oriented our choice. Children aged 5–15 years constitute also an important malaria infectious reservoir and a major contributor to maintaining malaria transmission within communities (Walldorf et al., 2015; Gonçalves et al., 2017). Children aged 5–15 years are not considered a high risk group for malaria and there is no control strategies that specifically address this particular population. In most countries these children can be reached at schools. We were able to mobilize the teachers from the Sirakoro-Meguetana schools and they accepted to contribute to children follow up and referral when required. Added to evidence from previous studies (Stevenson et al., 2013; Mphwatiwa et al., 2017), our study support the possibility to implement school-based malaria control activities. Having in perspective malaria elimination, school-based interventions bear a strong potential to help increase target population coverage with proven malaria control interventions.

SMC with SP-AQ has selected for molecular markers associated with resistance to SP in the children receiving SMC, in a Malian study. However a similar selective effect was not observed in general population. And the study did not find selection of molecular markers associated with resistance to AQ (Maiga et al., 2016). AQ has been used for decades in treating uncomplicated malaria in Africa. Contrary to chloroquine, a structural related drug abandoned due to high frequency of resistance, AQ is still effective in most West African countries (Olliaro and Mussano, 2003). A recent report of an increase in prevalence of parasites resistant *in vitro* to AQ, is a source of concern (Diawara et al., 2017). However, AQ is a synergistic companion drug to AS (Holmgren et al., 2007) and ASAQ has demonstrated sustained efficacy in the treatment of uncomplicated malaria (Fröberg et al., 2012). Since resistance to AS has emerged in Southeast Asia (Dondorp et al., 2009), several surveillance studies have been set up in Africa at sentinel sites to closely monitor the evolution of clinical efficacy of AS and the prevalence of molecular markers associated with delayed parasite clearance. No resistance to AS has been described in Africa (Menard et al., 2016).

ASAQ appears as an interesting alternative for SMC in areas with seasonal malaria transmission. Others artemisinin-based combination therapies (ACT) such as Dihydroartemisinin-Piperaquine (DHA-PQ) have been tested for SMC (Permala et al., 2017). DHA-PQ is being considered for mass treatment and for intermittent treatment during pregnancy. These highly efficacious ACTs have the potential to exert a powerful impact on malaria transmission in Sahelian areas and get close to malaria elimination. In populations where SMC is applied, incidence of clinical malaria drops substantially. We advocate that the most efficacious and well tolerated drugs be used for intermittent preventive treatment approaches. Such preventive programs must include a strong component of monitoring resistance to all drugs used.

The study showed an 86% protective efficacy against *P*. *falciparum* clinical malaria and a reduction of 67% in the prevalence of malaria infection. However no impact on the prevalence of anemia was observed. Several studies have assessed the impact of SMC in school aged children using various drug regimens (Barger et al., 2009; Nankabirwa et al., 2010; Nankabirwa et al., 2014b; Clarke et al., 2008; Clarke et al., 2017). ACTs regimens were the most efficacious against malaria disease and infection when compared with no artemisinine-based combinations therapies. The absence of effect on anemia could be explained by the lack of power to detect small difference in the prevalence of anemia, the presence of others factors than malaria that contribute to the etiology of anemia in school-aged children. Finally, the study population seems to be rather wealthy, with the presence of electricity in most houses and the level of literacy of parents.

The study duration was short, covering partially only one transmission season. Therefore we could not appreciate the potential rebound effect in the following transmission season. A trial has shown in Mali the absence of rebound the second year after SMC using SP-AQ (Dicko et al., 2011b).

Safety profile after ASAQ oral intake was acceptable. Most children had abdominal pain and headaches. ASAQ was administered at school, in the morning and without accompanying food to children. This may explain why so many had abdominal pain. These adverse events were reported after all three rounds of ASAQ administration. But no increase in frequency or intensity of adverse events was noticed. Besides one case, overall ASAQ was well accepted by children and their parents. The case was a child that had moderate abdominal pain and vomiting after the first round. The parent decided to withdraw from the study. The child remains in the study for safety follow up and received no additional dose of ASAQ. Most adverse events reported were of mild intensity and were almost all resolved without specific treatment after 4 days.

## 5 Conclusion

This open label controlled randomized trial showed that four rounds of ASAQ administered to school-aged children (6–15 years old) in Mali reduce the risk of clinical malaria by 86%, and the prevalence of malaria infection by 67%. ASAQ was a highly effective combination therapy with the potential to replace SP-AQ in case of spread of resistance to one of SP components. The study also showed that SMC delivery at schools with schools teachers as partners is feasible. This is a potential strategic approach to achieve universal coverage in view of malaria elimination in areas of Sahel where malaria transmission is seasonal.

## Conflicts of interest

None.

## Funding

The study drug ASAQ was donated by Guilin Pharmaceutical Co., Ltd.

The trial was funded by the Centre National de la Recherche Scientifique et Technologique (CNRST), the Malian National Center for Scientific Research and Technology, award number Programme de Recherche CNRST 10. The study was conducted within the African Network for Drugs and Diagnostics (ANDI) Center of Excellence for Clinical Development of Malaria Products.

The funding source had no role in study design, the collection, analysis and interpretation of data; the writing of the report; and in the decision to submit the article for publication.

## Authors' contributions

MAT, AKK, OKD, conceived and designed the study. AKK, BT, ED, SN, BD conducted the study and collected data. MSS analyzed the data. MAT, AKK, MSS, BT and OKD wrote the manuscript. All authors read and approved the final manuscript.
